# EdgeMap: An Optimized Mapping Toolchain for Spiking Neural Network in Edge Computing

**DOI:** 10.3390/s23146548

**Published:** 2023-07-20

**Authors:** Jianwei Xue, Lisheng Xie, Faquan Chen, Liangshun Wu, Qingyang Tian, Yifan Zhou, Rendong Ying, Peilin Liu

**Affiliations:** School of Electronic and Electrical Engineering, Shanghai Jiao Tong University, Shanghai 200240, China; xuejw2019@sjtu.edu.cn (J.X.); 15955192071@sjtu.edu.cn (L.X.); fqchen1998@sjtu.edu.cn (F.C.); wuliangshun@sjtu.edu.cn (L.W.); tqyang_sjtu@sjtu.edu.cn (Q.T.); yifanzhou-0713@sjtu.edu.cn (Y.Z.); rdying@sjtu.edu.cn (R.Y.)

**Keywords:** edge computing, spiking neural networks, neuromorphic hardware, mapping

## Abstract

Spiking neural networks (SNNs) have attracted considerable attention as third-generation artificial neural networks, known for their powerful, intelligent features and energy-efficiency advantages. These characteristics render them ideally suited for edge computing scenarios. Nevertheless, the current mapping schemes for deploying SNNs onto neuromorphic hardware face limitations such as extended execution times, low throughput, and insufficient consideration of energy consumption and connectivity, which undermine their suitability for edge computing applications. To address these challenges, we introduce EdgeMap, an optimized mapping toolchain specifically designed for deploying SNNs onto edge devices without compromising performance. EdgeMap consists of two main stages. The first stage involves partitioning the SNN graph into small neuron clusters based on the streaming graph partition algorithm, with the sizes of neuron clusters limited by the physical neuron cores. In the subsequent mapping stage, we adopt a multi-objective optimization algorithm specifically geared towards mitigating energy costs and communication costs for efficient deployment. EdgeMap—evaluated across four typical SNN applications—substantially outperforms other state-of-the-art mapping schemes. The performance improvements include a reduction in average latency by up to 19.8%, energy consumption by 57%, and communication cost by 58%. Moreover, EdgeMap exhibits an impressive enhancement in execution time by a factor of 1225.44×, alongside a throughput increase of up to 4.02×. These results highlight EdgeMap’s efficiency and effectiveness, emphasizing its utility for deploying SNN applications in edge computing scenarios.

## 1. Introduction

The growing popularity of edge devices, such as wearable electronics, the Internet of Things devices, industrial machinery, and smartphones connected to the internet, has been driving the development of intelligent applications [1]. Edge computing has emerged as a promising approach to optimize overall system performance by reducing latency and conserving bandwidth through processing data closer to the source rather than relying on centralized nodes. As a result, there is a growing interest in endowing edge computing with greater intelligence [2].

Spiking neural networks (SNNs) demonstrate significant potential in providing edge devices with intelligent capabilities due to their low energy consumption and powerful biomimetic intelligence features [3]. An increasing number of SNNs have exhibited exceptional computational performance in various edge computing applications, including object recognition [4], speech recognition [5], and robotics control [6]. Through the utilization of SNNs, edge devices can be transformed into more intelligent nodes capable of executing complex intelligent applications with enhanced accuracy and efficiency.

Recently, neuromorphic hardware designs, such as TrueNorth [7], Loihi [8], and SpiNNaker [9], have recently emerged. These designs feature multiple interconnected neuromorphic cores (NCs) through a network-on-a-chip (NoC), enabling the energy-efficient and high-performance execution of SNNs [10]. To bridge the gap between SNNs and hardware implementation, various mapping schemes have been proposed [11,12,13]. These schemes typically involve two stages: partitioning and mapping. During the partitioning stage, the computation graph of SNN neurons is divided into clusters under the constraints of the neuromorphic hardware. The mapping stage then places these clusters onto physical neuron cores to achieve different optimization objectives.

However, while these mapping schemes enhance the performance, scalability, and accuracy of SNNs on neuromorphic hardware, they primarily focus on neuromorphic hardware deployed at the server end at present. This approach assumes a stable environment and unlimited computational resources, which is not reflective of edge computing scenarios. Implementing SNNs on compact edge devices, thus, presents unique challenges, making it a more complex and rigorous task than server-end deployments. These challenges may include:

- Energy constraints: Edge computing devices, typically standalone units, operate under stringent energy constraints, making power efficiency a critical factor in their operation. Different mapping schemes can lead to substantial disparities in energy consumption, significantly affecting the performance and viability of these devices. These realities underscore the urgent need for mapping schemes that thoroughly consider energy efficiency.
- Real-time processing: Many edge computing applications require real-time or near-real-time processing, necessitating low latency and efficient network communication. However, achieving these performance characteristics without compromising other essential attributes, such as energy efficiency, is a challenging balancing issue.
- Complex environments: Edge computing scenarios are inherently complex and diverse, often with varying constraints and requirements. Edge environments, unlike server-end environments, can have much more diverse and demanding constraints and requirements.

Drawing upon the aforementioned challenges, this paper aims to explore the optimal mapping toolchain for SNN applications under multiple edge computing environments. We develop a partitioning algorithm for various SNNs to enable rapid and resource-efficient partitioning on edge devices. The mapping optimization strategy is then applied to meet edge computing requirements. The main contribution of this paper is to provide a more efficient and flexible deployment of neuromorphic computing in edge computing applications while also improving the overall system performance.

- We propose a novel partitioning method inspired by the streaming-based approach [14]. This method efficiently partition SNNs into multiple neuron clusters, significantly reducing partitioning time while minimizing the number of spikes between clusters. Importantly, our approach takes into account the fan-in and fan-out limitations of neuromorphic hardware, ensuring compatibility with edge device constraints.
- We propose a multi-objective optimization method to place neuron clusters onto neuromorphic devices. This method addresses the diverse challenges in edge computing, including bandwidth limitations, communication costs, and energy consumption. Our approach, while successfully reducing energy consumption and latency, crucially enhances the overall performance of neuromorphic computation in edge environments.
- We propose a versatile mapping toolchain suitable for a wide range of neuromorphic edge devices. By conducting extensive evaluations of various SNNs and scales on NoC-based hardware, we demonstrate significant enhancements in edge computing performance. In addition, our toolchain can accommodate the diverse constraints and requirements of different edge computing scenarios.

This paper is organized as follows: Section 2 provides a review of the related work. In Section 3, we discuss the necessary background for understanding the subsequent sections. Section 4 delves into the design methodology of EdgeMap. Section 5 outlines the setup used for our evaluations. In Section 6, we present a detailed account of our experimental results. Finally, in Section 7, we conclude this paper.

## 2. Related Work

Recently, SNNs have garnered interest due to their energy efficiency and computational capabilities [3]. However, executing SNNs on neuromorphic hardware is challenging because of their complex connections and hardware limitations. Many efforts have been made to bridge this gap, with current SNN mapping schemes generally falling into three categories: dedicated hardware mapping, general mapping for crossbar-based hardware, and other flexible mapping schemes.

The first category involves dedicated hardware design schemes specifically tailored for specialized SNN solutions. Examples of such mapping schemes include SentryOS [15] for μBrain [16], the Corelet toolchain [17] for TrueNorth [7], and the LCompiler [18] for Loihi [8]. These complete toolchains aim to optimize core utilization to the maximum extent. The SentryOS toolchain comprises a SentryC compiler, which partitions SNN network structures into multiple subnetworks, and a SentryRT real-time manager responsible for sorting and computing different subnetworks in real-time. Corelet, a dedicated programmable toolchain for TrueNorth, encapsulates biological details and neuron complexity before proposing an object-oriented Corelet language to compose and then execute SNNs. The LCompiler framework maps SNNs onto Loihi neuromorphic hardware by presenting a data flow graph with logical entities describing an SNN instance, such as compartments, synapses, input maps, output axons, and synaptic traces. The framework first transforms SNNs into microcodes and translates SNN topology into a connection matrix. Next, it employs a greedy algorithm to map logical entities to hardware before generating the bitstream for Loihi cores. In the study by Wang et al. [19], they presented an innovative mapping method specific to the Tianjic neuromorphic chip [20]. This approach comprises two stages: logical mapping and physical mapping. In the logical mapping stage, the authors introduce a closed-loop mapping strategy that employs an asynchronous 4D model partition. For the physical mapping stage, a Hamilton loop algorithm is applied. The advantage of this method is its ability to achieve high resource utilization and processing efficiency, providing an interesting perspective for the efficient deployment of neural networks on neuromorphic hardware. All of these mapping toolchains are designed for specific targets, restricting their universal applications. This restricts their adaptability for more general-purpose use.

The second category focuses on crossbar-based SNN topology schemes, which are more general mapping techniques. These schemes include state-of-the-art mapping techniques, such as NEUTRAMS [21], SpiNeMap [13], SNEAP [22], and DFSynthesizer [23]. NEUTRAMS is a co-design toolchain that partitions SNNs to meet hardware constraints and optimizes their mapping onto neuromorphic hardware. SpiNeMap adopts a greedy approach, loosely based on the Kernighan–Lin graph partitioning algorithm [24], to minimize inter-cluster spike communication, and it utilizes a PSO algorithm [25] to place each cluster on a physical core. SNEAP [22] uses the METIS graph partition algorithm to partition the SNN computation graph with the objective being the communication cost, then it uses a greedy algorithm to become the final mapping results. DFSynthesizer [21] is an end-to-end framework for mapping machine learning algorithms. It decomposes and partitions SNNs into clusters before exploiting the rich semantics of synchronous data flow graphs to explore different hardware constraints that influence mapping performance. These existing toolchains are designed mainly for cloud servers and do not consider the constraints of complex computing environments like edge computing. This could limit their applicability in resource-constrained edge scenarios where environmental factors come into play.

Various works have been proposed to address specific challenges in mapping schemes. For instance, Jin et al. [26] aimed to address the challenge of mapping large-scale SNNs onto neuromorphic hardware. Their approach, which employs a Hilbert curve and a force-directed algorithm to optimize the mapping stage, achieves remarkable time degradation when deploying large-scale SNNs. Similarly, Nair  [27] suggested mapping recurrent neural networks to in-memory computing neuromorphic chips by modifying the ANN to an RNN unit with an adaptive spiking neuron model and scaling it to fit the chip. Additionally, Migspike [28] was introduced to address reliability concerns resulting from the probability of accumulating faults, and it uses a node-level recovery mechanism for neuron failures by placing spare neurons into each neuron core, implemented through a mapping method for defective neurons.

In summary, various schemes have been proposed to optimize the mapping of neuromorphic hardware to achieve different optimization goals. To build upon these current mapping schemes, the objective of this paper is to introduce a novel mapping toolchain that is customized for complex edge computing scenarios with limited energy and computing resources that also require low latency.

## 3. Background and Motivation

This section begins with a brief overview of SNNs, emphasizing their unique attributes that facilitate highly efficient computational patterns and capabilities. Subsequently, we provide an introduction to edge computing, delving into the complexities of its inherent environments and the accompanying requisites.

### 3.1. A Brief Introduction to SNN

Inspired by mammalian brains, SNNs have become increasingly popular for use in AI applications due to their ability to process data in a more efficient manner than traditional neural networks. Moreover, SNNs are less prone to overfitting and are better at capturing nonlinear patterns, making them well-suited for a wide range of artificial intelligence applications [6,29,30].

**Figure 1:**
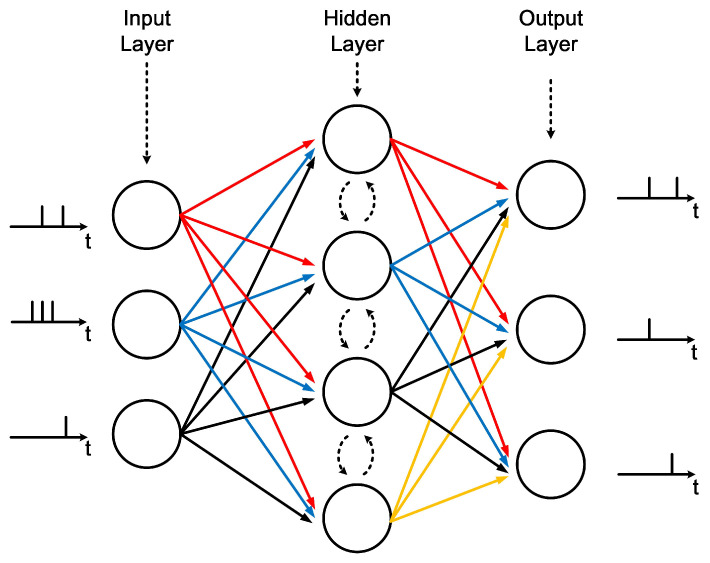
A three-layer SNN topology.

A typical and fundamental SNN model, as illustrated in Figure 1, can be represented as a directed graph consisting of a series of connected layers. The model might also incorporate inter-layer connections, enhancing the network’s connectivity and learning capabilities. Each neuron in an SNN is connected to a specific number of neurons, with adjustable connection weights, allowing the network to learn from data. During computation, each neuron in an SNN processes weighted stimulus spikes from neighbors, generating and transmitting spike outputs to output neurons. This iterative process continues until the output layer produces the final results.

In this paper, we focus primarily on the inference of SNNs rather than training. This is because the training process is generally performed offline through powerful cloud data centers and is typically delay-tolerant [31], which we do not consider in this paper.

### 3.2. Edge Computing

**Figure 2:**
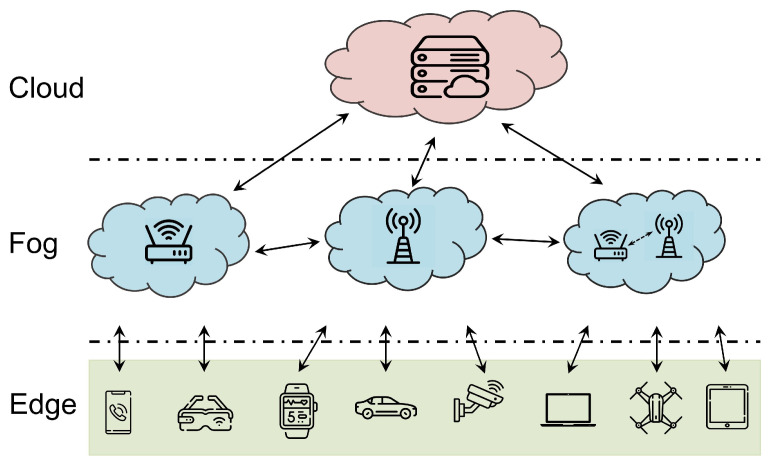
Example of the edge–fog–cloud architecture.

The edge–fog–cloud architecture, which has emerged as a popular paradigm in distributed computing, as depicted in Figure 2, has demonstrated its applicability across various domains for its significant advantages in managing computational resources and reducing communication latency [32]. This structure incorporates three layers: the edge, fog, and cloud layers, with the fog layer serving as a bridge between edge and cloud computing. Unlike traditional cloud computing, which predominantly depends on input data from edge devices such as images [33], speech [34], and videos [35], edge computing alleviates the burden on cloud computing centers by offering processing and storage services closer to end-users. However, progressing from the edge towards the cloud usually yields more sophisticated machine learning and data processing capabilities but introduces higher latency and communication overheads. The fog layer, featured in contemporary architectural designs, functions as a grid that mediates between the edge and the cloud, helping to balance the advantages and drawbacks of the other two layers. This layer addresses concerns associated with reliability, load balancing, communication overhead, and data sharing. Particularly, the fog layer acts as a data collector close to the edge layer and as a data filter close to the cloud layer, streamlining the data flow in near real-time for efficient processing, compression, and transformation.

Despite the potential of the edge–fog–cloud architecture, the increasing demands of edge devices exert substantial pressure on the cloud and fog layers. This situation underscores the need for enhancing intelligence at the edge. A significant step towards this goal is the exploration and implementation of SNNs in edge computing. However, this integration comes with unique challenges. While SNNs offer high computational efficiency and biological realism, their inherent characteristics, such as temporal dynamics and spike-based communication, complicate their deployment. Limited hardware resources and the complexity of neuron connections pose additional difficulties in mapping SNNs onto neuromorphic hardware. The balance between enhancing intelligence for edge computing applications and navigating the constraints of edge devices forms the focal point of our discussion in this paper.

In addition, the adoption of edge computing itself comes with a unique set of challenges, primarily due to the constrained resources and the need for real-time data analysis and decision-making. The struggle is particularly evident in applications like IoT devices [36], autonomous vehicles [37], and smart city infrastructure [38]. To balance the algorithm accuracy, energy consumption and communication efficiency amidst diverse devices and complex scenarios are significant difficulties. Individual edge computing scenarios with limited computational power were often unable to cope with large-scale data processing. Conversely, collaborative edge computing scenarios require efficient coordination schemes to manage variations in computational capabilities and device numbers. Consequently, conventional practices, such as deploying AI models or transferring data to the cloud for processing, prove inadequate for applications demanding real-time, high-intelligence performance.

These constraints and the limitations of current solutions underscore the necessity of a novel approach. It is within this context that this work introduces a mapping toolchain specifically designed for deploying SNNs on edge computing devices. This innovation aims to bridge the gap between SNNs and edge computing, with an emphasis on low-latency and high-intelligence performance.

## 4. Framework and Design Method

In this section, we present a comprehensive overview of EdgeMap, an optimized mapping toolchain specifically designed for SNNs in edge computing environments, which addresses the inherent limitations and challenges associated with edge computing scenarios.

### 4.1. Framework Overview

**Figure 3:**
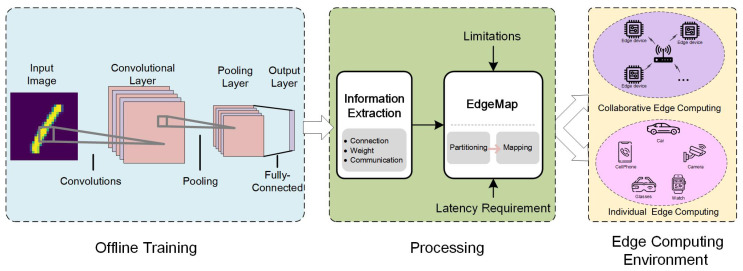
A high-level overview of EdgeMap.

**Figure 4:**
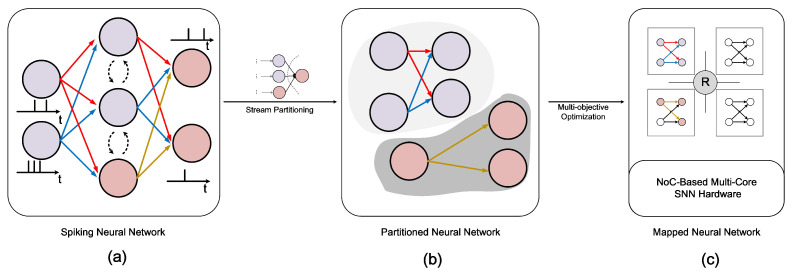
Flowchart of the mapping. (**a**) The connection of SNNs. (**b**) The partitioned neuron clusters. (**c**) Neuron cluster to NCs mapping.

The EdgeMap framework aims to enhance the performance of SNNs on neuromorphic hardware in edge computing scenarios. Current mapping approaches primarily focus on the forward direction of neural network inference, as the learning phase can be latency-insensitive and conducted through the cloud. As illustrated in Figure 3, the EdgeMap toolchain deploys a specific neural network through the following stages:

- Offline training stage: The design and training of models with different network structures are crucial, considering the diverse application scenarios in edge computing. These structures must meet varied accuracy and scale requirements. The training process can be implemented using frameworks like Nengo [39], CARLsim [40], and Brain2 [41]. For clarity, we use the commonly adopted convolutional SNNs for recognition applications in Figure 3, primarily used for classification tasks [42].
- Processing stage: As shown in Figure 4, this stage is pivotal to the entire process. We start by extracting information from trained SNNs, such as neuron connection structure, weight information, and spike communication details. The SNN topology is represented as a computing graph, as depicted in Figure 4a. Subsequently, we divide the SNN into smaller neuron clusters for more manageable processing, demonstrated in Figure 4b. Finally, we map these partitioned neuron clusters onto the neuromorphic core, as illustrated in Figure 4c. This process forms the core function of our EdgeMap toolchain. It optimizes hardware resource utilization and ensures computational efficiency.
- Edge computing stage: In this part, we briefly introduce two edge computing modes: individual edge computing scenarios and collaborative edge computing scenarios. The individual edge computing scenario involves a variety of computing modes and different types of devices. As for collaborative edge computing scenarios, multiple edge devices cooperate via network connections to accomplish more computing require tasks.

Based on the above introduction, the EdgeMap toolchain covers the entire process from training and processing to edge computing, adapting to the demands of various scenarios, and effectively improving the performance of SNNs on neuromorphic hardware in edge computing contexts.

### 4.2. Neuromorphic Edge Computing Hardware Model

In this paper, we focus on the deployment of SNNs on edge devices in two scenarios: individual edge computing and collaborative edge computing. The individual edge computing suits situations with a relatively small SNN scale or sufficient computing resources on a single edge device. Conversely, when a single edge device’s computing resources are insufficient, collaborative edge computing becomes necessary, requiring multiple devices for computations.

**Figure 5:**
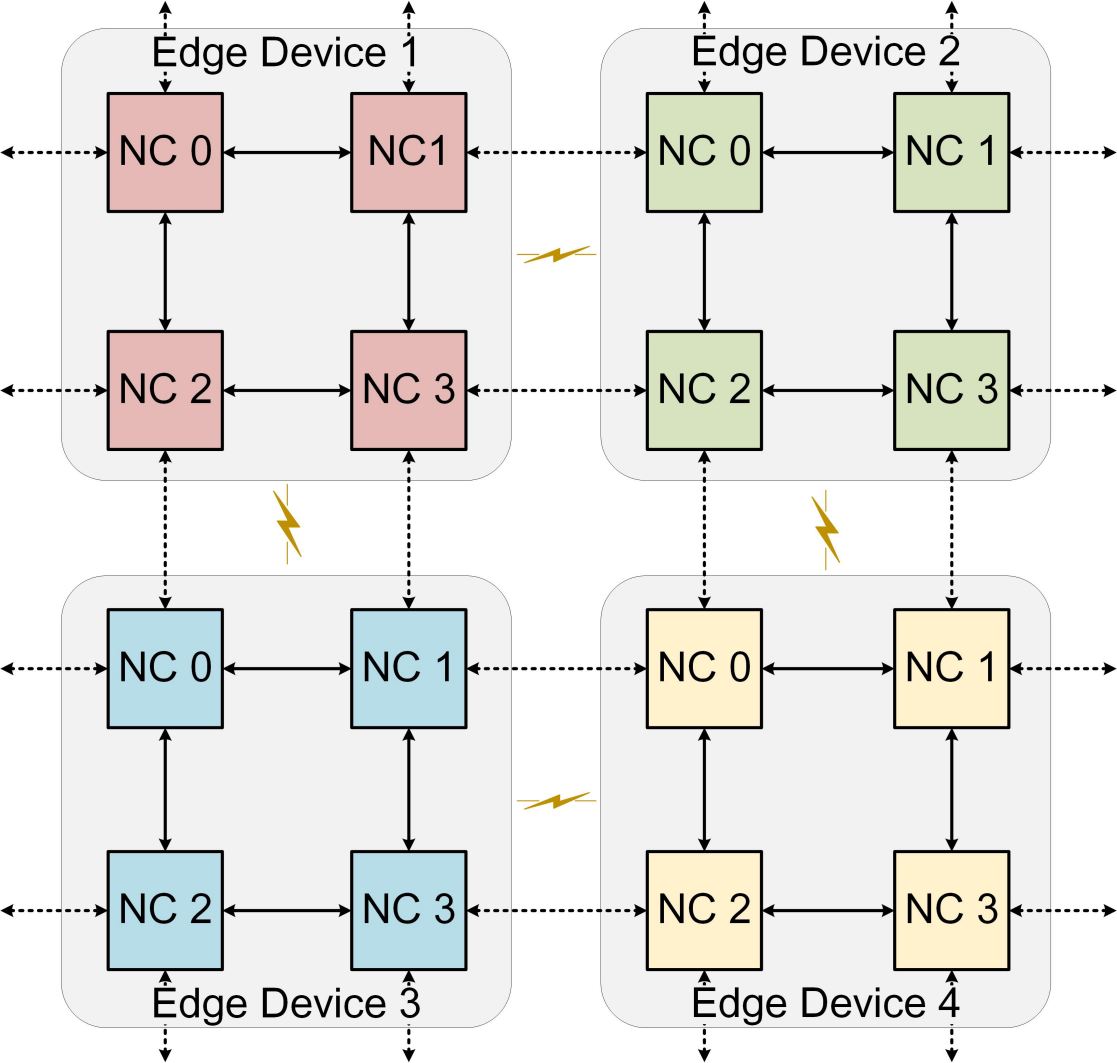
An illustration of the neuromorphic edge computing hardware model.

To provide a clearer understanding of the edge computing modes, we devise a simplified hardware model as depicted in Figure 5. For the sake of clarity, we assume that each edge device consists of four neuromorphic cores interconnected through a 2D mesh. Although actual devices may have more cores, our model seeks to effectively clarify the system’s fundamental architecture. Additionally, to emulate collaborative edge computing, we model four edge devices as interconnected via a mesh topology. The dashed lines in the figure represent the communication pathways between devices within a cooperative edge computing environment, with the fog layer being the primary facilitator for such interactions. For the purposes of our study, we make the following assumptions:

- The spike communication latency and energy consumption between devices are equivalent to those within the device’s neuromorphic cores.
- The energy required for spike processing is uniformly distributed across all edge devices.
- The NCs across different devices possess equal computational capacity, including identical quantities of neurons and synapses.

By setting these assumptions, we establish a general framework to study the deployment of SNNs under various device and application scenarios. This enables us to gain a better understanding of the key aspects of cooperative edge computing and provides a more comprehensive evaluation of the performance of different mapping algorithms in an edge computing environment.

### 4.3. Streaming-Based SNN Partition

After training the SNNs, we use the resulting connectivity information to transform the SNN models into a computing graph G={V,E}, where *V* is the set of neurons and *E* is the set of synapses. We then partition *V* into clusters $C=(C_{1},C_{2},\cdots ,C_{k})$ to accommodate the constraints of neuromorphic hardware.

The partitioning process, as illustrated in Figure 4b and referred to as SNN partition, aims to partition SNNs into smaller neuron clusters. Our proposed SNN partition algorithm employs a streaming-based graph partitioning strategy, systematically assigning neurons to various clusters using a tailored gain function *g* to optimize both inter and intra-cluster communication costs based on the number of spikes transmitted.

In the context of our proposed SNN partition algorithm, *k* denotes the total number of clusters, which is no more than the number of NCs or edge devices. The gain function g(C) is the sum of all cluster gains $h(C_{i})$, where $h(C_{i})$ is the gain of cluster $C_{i}$. The term $\sum_{e\in e(X,Y)}w\left(e\right)$ represents the total number of spikes transmitted between *X* and *Y*. We use a specific convex increasing function $c\left(x\right)=\alpha x^{\gamma },\alpha \geq 0,\gamma \geq 1$ to measure the cost of a cluster, with $c\left(x\right)=x^{2}$ in our implementation, which quantifies the cost associated with the size of the cluster $|C_{i}|$. This approach allows us to balance the trade-off between the communication cost and cluster size, contributing to the efficient utilization of the NCs or edge devices.

(1) $$\begin{array}{cc} g(C) & =\sum_{i=1}^{k}h\left(C_{i}\right)\\ \; & =\sum_{i=1}^{k}\sum_{e\in e(C_{i},C_{i})}w\left(e\right)-c(|C_{i}\left|\right)\\ \; & =\left(\sum_{e\in E}w\left(e\right)-\sum_{i=1}^{k}\sum_{e\in e(C_{i},V\setminus C_{i})}w\left(e\right)\right)-\sum_{i=1}^{k}c(|C_{i}\left|\right) \end{array}$$

**Figure 6:**
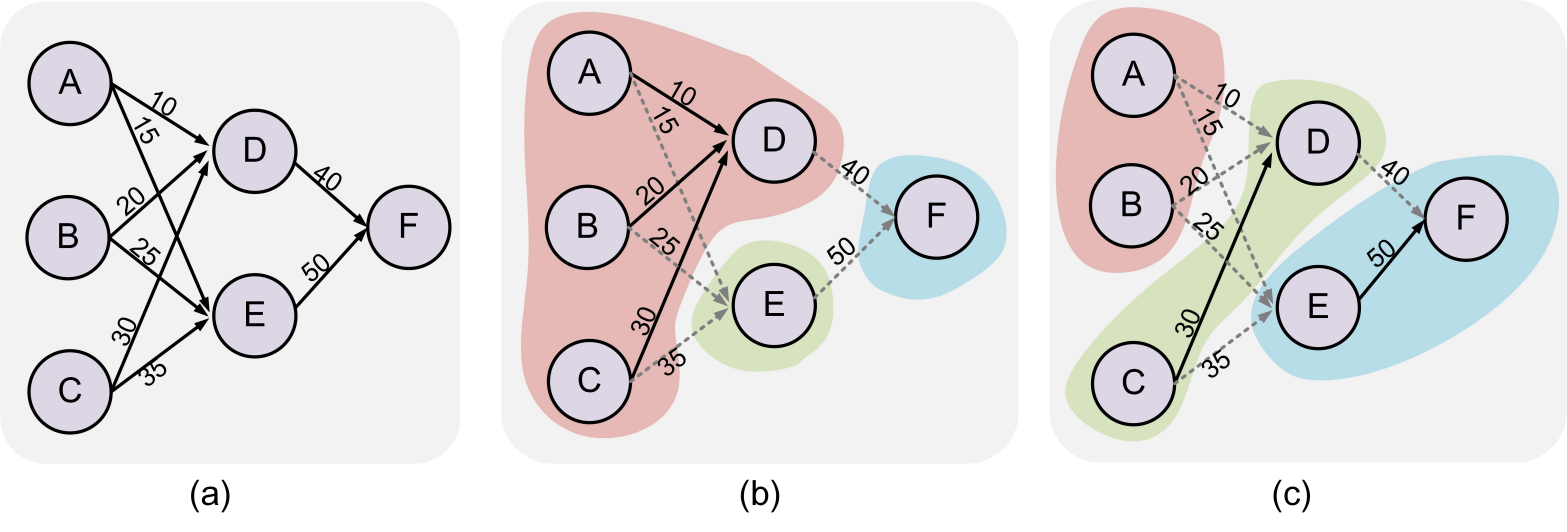
Exhibition of partition strategies and corresponding cost analysis: (**a**) original SNN graph displaying the number of spikes, (**b**) partition result producing 3 clusters consisting of 4 neurons, 1 neuron, and 1 neuron, respectively; (**c**) alternative partition result resulting in 3 clusters, each containing 2 neurons.

To illustrate the partitioning cost more clearly, we present a straightforward example of our proposed method in Figure 6. Subfigure (a) showcases the original SNN graph with the number of spikes indicated on each edge. Two distinct partition schemes are depicted in subfigures (b) and (c). Neurons that are colored the same belong to the same cluster. The gray dashed lines denote the connection edges that need partitioning, contributing to the communication cost of the neuron cluster. Scheme (b) results in a communication cost of 165 and a cluster size cost of 18, yielding a total gain of 183. On the other hand, scheme (c) incurs a communication cost of 155 and a cluster size cost of 12, resulting in a total gain of 167. Therefore, scheme (c) achieves a superior gain *g*, indicating its greater efficiency as a partitioning scheme.

In the partitioning process, a greedy approach is adopted. The gain of assigning neuron $v_{i}$ to cluster $C_{j}$ is defined as $\delta g(v_{i},C_{j})$. The objective is to allocate each neuron $v_{i}$ to a cluster $C_{f}$ that maximizes $\delta g(v_{i},C_{f})$, for all $C_{i}\in C$.

(2) $$\begin{array}{cc} \delta g(v_{i},C_{j})= & g(C_{1},\cdots ,C_{j}\cup v_{i},\cdots ,C_{m},\cdots ,C_{k})\\ \; & -g(C_{1},\cdots ,C_{j},\cdots ,C_{m},\cdots ,C_{k}) \end{array}$$

In conclusion, our SNN partition algorithm provides an effective means of partitioning the SNN computation graph across multiple neuromorphic devices. By minimizing communication costs and balancing the size of each cluster, it ensures optimal performance while adhering to the device’s constraints. This carefully calibrated approach leads to improvements in both computational efficiency and communication overhead, making it particularly beneficial for applications, such as real-time data processing and decision-making tasks. Examples of such applications include autonomous vehicles, smart home systems, and various edge computing scenarios.

Delving into the specifics of our method, the SNN partitioning algorithm we propose is detailed in Algorithm 1. Initially, *k* empty clusters are created, where k=⌈|V|/N⌉, and *N* is the maximum number of neurons a core can accommodate. Each neuron $v_{i}$ is then allocated to its optimal cluster $C_{final}$, considering the hardware limit of crossbars. The canAllocate($v_{i}$, $C_{j}$) function checks the feasibility of allocating a neuron $v_{i}$ to a cluster $v_{i}$, and addVertexToCluster($v_{i}$, $C_{final}$) subsequently adds the neuron to its assigned cluster.

**Algorithm 1:** Flow-based SNN partition

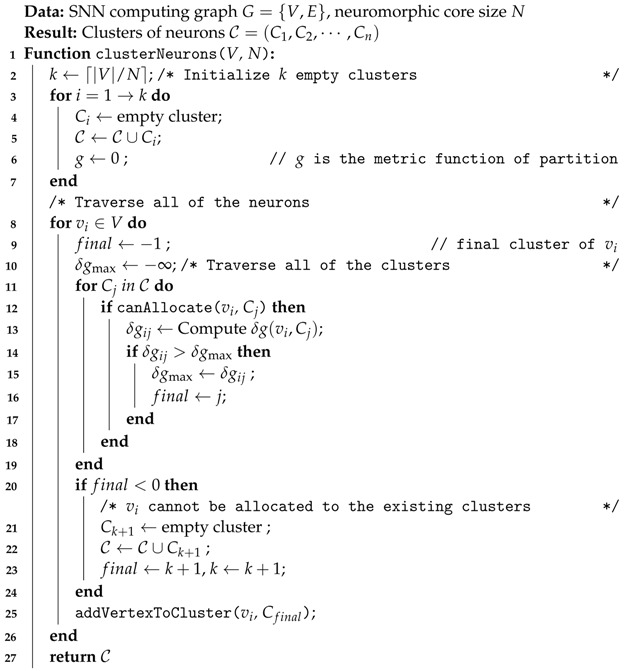



In the execution of our algorithm, we partition the SNN computation graph into manageable clusters for individual neuromorphic devices. Optimizing the gain function *g* reduces the communication overhead and ensures efficient utilization of hardware resources. The algorithm selects the cluster with the largest gain δg for each neuron $v_{i}$, maintaining a time complexity of O(|V||C|), linear to the size of the neural network and the cluster set.

### 4.4. Multi-Objective Optimization Mapping

**Figure 7:**
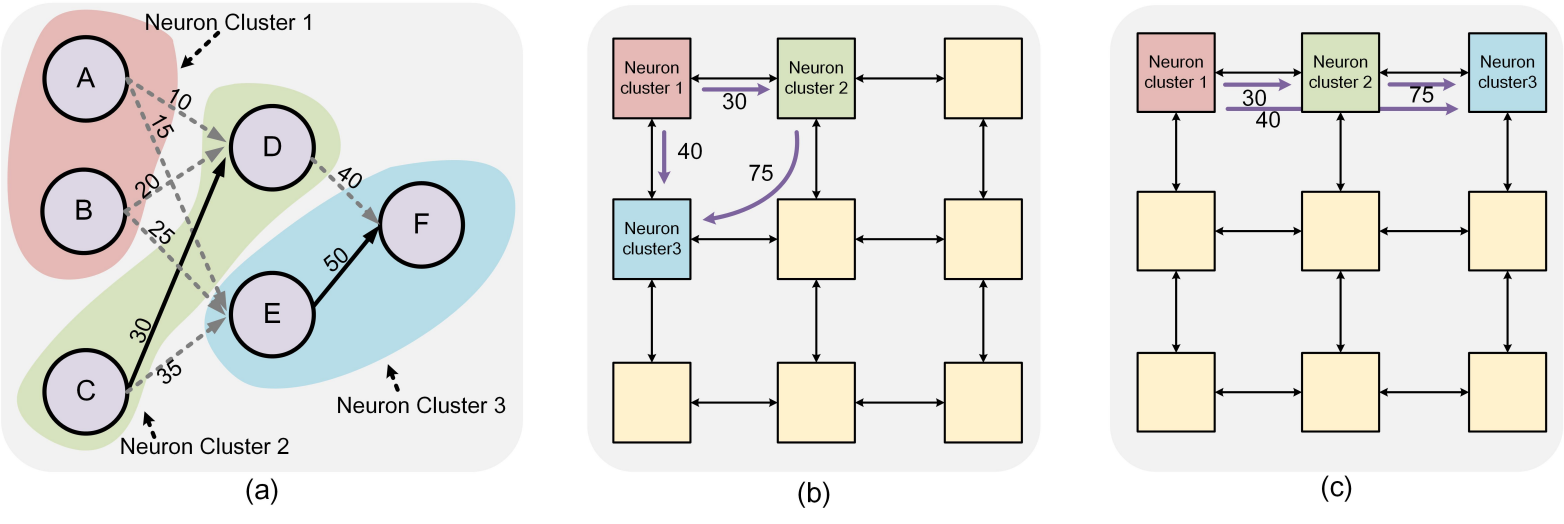
Demonstration of neuron cluster mapping strategies for NCs: (**a**) partitioned neuron clusters, (**b**) mapping scheme with low congestion, and (**c**) mapping scheme with high congestion.

In this paper, we operate under the assumption that a sufficient number of physical cores are available on edge devices for the parallel processing of all neurons in the SNNs. The mapping stage, which is vital for multi-core collaborative computing, especially in edge scenarios, assigns partitioned neuron clusters to corresponding NCs as depicted in Figure 4c. As shown in Figure 7, the choice of mapping schemes can significantly influence energy consumption, average latency, and hop count. In Figure 7a, we display the neuron clusters post-partitioning. Subfigures (b) and (c) present two different mapping schemes. In scheme (b), the maximum communication throughput is 75 (spike). On the other hand, scheme (c) increases the maximum communication throughput to 115 (total spike from 2 to 3 and 1 to 3). However, it also results in higher congestion in the cluster 2 node, leading to increased latency. Therefore, the selection of the mapping scheme should strike a balance between these various factors to achieve the most efficient outcome.

To effectively simulate the communication between NoC-based multi-core or multi-device neuromorphic computing, we developed a Noxim-based simulator [43]. This tool not only provides an accurate model for communication within NoC-based multi-core neuromorphic hardware but also supports various edge computing scenarios, facilitating collaboration among multiple devices.

Mapping definition: Placing the neuron cluster set $C=(C_{1},C_{2},\cdots ,C_{k})$ onto the physical computing hardware with a set of hardware computing cores $P=(P_{1},P_{2},\cdots ,P_{k})$. This association can be denoted as C→P and is detailed through a logical matrix, $m_{ij}$, belonging to ${\left\{0,1\right\}}^{k\times k}$. The element $m_{ij}$ within the matrix is defined as follows:

(3) $$m_{ij}=\left\{\begin{array}{cc} 1 & \;\mathrm{if}\;\mathrm{partition}\;C_{i}\in C\;\mathrm{is}\;\mathrm{mapped}\;\mathrm{to}\;\mathrm{device}\;P_{j}\in P\\ 0 & \;\mathrm{otherwise}\; \end{array}\right.$$

and the mapping constraints are the following.

(1) A neuron cluster can be mapped to only one computing device, thereby establishing a unique mapping relationship. Mathematically, this constraint can be expressed as follows:
  (4) $$\sum_{j}m_{ij}=1\;\forall i$$
(2) A computing device can accommodate, at most, one neuron cluster, ensuring exclusivity of the mapping process. This can be formally stated as follows:
  (5) $$\sum_{i}m_{ij}\leq 1\;\forall j$$

In this paper, we primarily aim to maximize the performance of SNNs on edge devices while bolstering their energy efficiency. These objectives are primarily driven by the need to satisfy the stringent energy constraints and meet real-time processing demands, which are discussed in Section 1. This is under the assumption provided in Section 4.2 that the spike processing speed and energy consumption are constant. The real-time performance and energy consumption are typically influenced by the mapping results in the context of NCs. It should be acknowledged that optimizing one objective may degrade others, leading to a multi-objective optimization problem. To address this, we consider several optimization objectives concurrently, intending to meet the diverse resource requirements that are characteristic of edge computing scenarios. The following are some of the critical objectives:

(1) Total energy consumption: This objective captures the cumulative energy consumed by all spikes on the hardware, providing a direct reflection of the energy consumption. It is computed as
  (6) $$E=\sum_{i=1}^{N_{s}}\left[\left(h_{i}-1\right)*e_{w}+h_{i}*e_{s}\right]$$
  where $N_{s}$ is the total number of spikes, $e_{w}$ and $e_{s}$ represent the energy consumption of spikes on the communication and cores, respectively.
(2) Communication cost: This is the total communication count of the NCs, acting as a reflection of the system’s communication efficiency. This metric has substantial implications for performance and power consumption. It is computed as
  (7) $$M=\sum_{i=1}^{k}\sum_{j=i}^{k}s_{ij}*h_{ij}$$
  where *k* is the number of clusters, $s_{ij}$ is the number of spikes between clusters *i* and *j*, and $h_{ij}$ is the hop distance between clusters *i* and *j* (Manhattan distance).
(3) Throughput: This metric represents the maximum number of spikes traveling on a link between any two neighboring clusters. As a pivotal metric directly influencing the system’s performance, it is computed as follows:
  (8) $$\begin{array}{cc} Throughput & =\max Load_{ij},1\leq i<j\leq k,\left|i-j\right|=1\\ Load_{ij} & =\mathrm{Total}\;\mathrm{spikes}\;\mathrm{through}\;channel_{ij} \end{array}$$
  where $Load_{ij}$ indicates the total number of spikes transmitted between clusters *i* and *j*.
(4) Average Hop: This metric offers insights into the efficiency of communication and overall performance. It denotes the average number of hops a spike requires to travel between neuron clusters, which mirrors the communication efficiency of the edge computing system. It is computed as follows:
  (9) $$H_{av}=M/\sum_{j=i}^{k}s_{ij}$$
  where *M* is the total communication cost from Equation (7).
(5) Average latency: This metric represents the average delay experienced by spikes on the NoC. The latency is critical to system responsiveness and the real-time nature of inference computations, particularly in edge computing applications requiring swift responses. It can be computed as follows:
  (10) $$E=\sum_{i=1}^{N_{s}}\left[\left(h_{i}-1\right)*l_{w}+h_{i}*l_{s}\right]$$
  where $h_{i}$ is the hop count for a spike from source to destination, $l_{w}$ is the communication delay, and $l_{s}$ is the processing delay of the hops.
(6) Average congestion: This metric represents the average communication pattern of the NoC, which is the connection network in the edge scenarios. It is especially critical for applications demanding real-time responses. It is computed as follows:
  (11) $$M_{ac}=\sum_{(x,y)\in S}Con\left(x,y\right)/N$$
  where Con(x,y) computes the congestion in the router, with coordinates of (x,y). It is computed as follows:
  (12) $$Con\left(x,y\right)=\sum_{e_{i,j}\in E_{P}}\left(w_{P}\left(e_{i,j}\right)\right.\left.*Expe\left(x,y,P\left(c_{i}\right),P\left(c_{j}\right)\right)\right),$$
  where Expe is the function that computes the expected value of the number of spikes that pass through the coordinates [26].

To tackle the complexities of these scenarios, we propose a multi-objective optimization algorithm based on NSGA-II [44], tailored specifically to edge computing applications where energy consumption and communication costs are vital factors. The optimization target, designed to simultaneously minimize the total communication costs and energy costs, is expressed in Equation (13).

(13) $$\underset{x}{\mathrm{argmin}}\left(M,E\right),s.t.\;x\;\mathrm{is}\;a\;\mathrm{valid}\;\mathrm{mapping}\;\mathrm{scheme}.$$

In order to find the best optimization solution, Algorithm 2 is used to optimize Equation (13). Firstly, we initialize the population with input size *N* (line1∼5). A quick non-dominated sorting is then performed on the initial population P (line 6). The offspring are generated using *P* (line 7) by selection, crossover, and mutation. We then iterate for several rounds until the convergence criteria are met (line 8∼19). In each round, we first perform the parent–child merge, then perform the fast non-dominated sorting. The top best *N* individuals are selected and then generate offspring for the next round based on these *N* individuals. Once the convergence criteria are met, individuals with rank 0 (non-dominated solutions) are filtered from the population (P) and added to the non-dominated (ND) population (line 20∼25). The non-dominated (ND) population is finally returned (line 26).

**Algorithm 2:** Multi-objective optimized mapping for edge computing

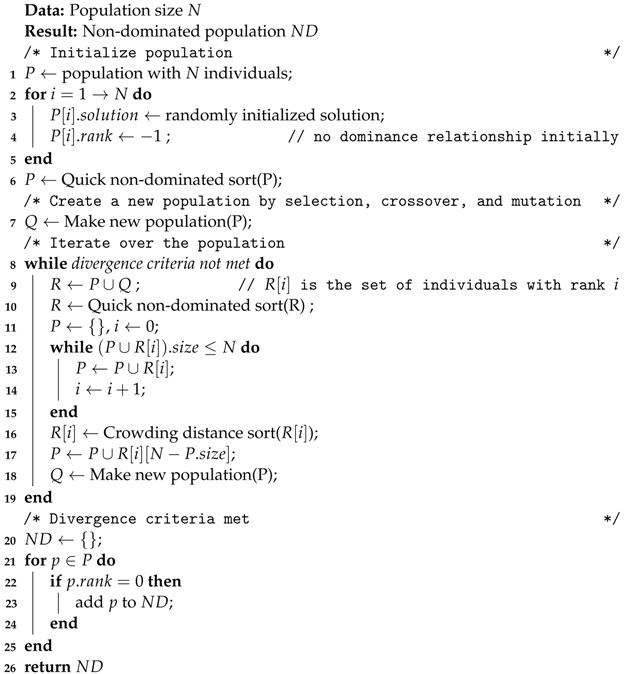



Upon obtaining the Pareto solution space through Algorithm 2, a location placement algorithm is needed to acquire the solution space, the task of which is performed by Algorithm 3. The goal of this algorithm is to convert each solution $Y_{i}$ from Algorithm 2 into a final mapping solution, represented as $X=(X_{1},X_{2},\cdots ,X_{k})$.

(14) $${\left(\frac{c_{1}}{\max c_{1}}\right)}^{2}+{\left(\frac{c_{2}}{\max c_{2}}\right)}^{2}$$

Let us recall that each $Y_{i}$ is an integer array of size *k*, where $1\leq y_{ij}\leq j+1$. The metric for selection is defined in Equation (14), where $\max c_{1}$ and $\max c_{2}$ represent the maximum values of the metrics computed during the execution of Algorithm 2.

(15) $$Y_{i}=\left(y_{i1},y_{i2},\cdots ,y_{ik}\right),1\leq i\leq \mathrm{population}\;\mathrm{size}$$

However, $Y_{i}$ does not represent the final mapping solution. Each final mapping solution, denoted by $X=(X_{1},X_{2},\cdots ,X_{k})$, is constructed from $Y_{i}$. Here, *X* is a sorted array ranging from 1 to *k*. The procedure for constructing *X* from $Y_{i}$ is detailed in Algorithm 3. The outcome of this procedure is that each cluster $C_{i}$ from C is mapped onto a physical core $P_{x_{i}}$ in P. This final mapping is essential for the optimization of the communication cost and energy cost in edge computing applications.

**Algorithm 3:** Procedure for the optimal mapping result

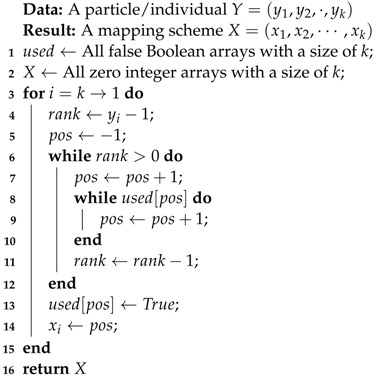



## 5. Evaluation Methodology

### 5.1. Experimental Environment

In this section, we describe the configuration of the edge computing environment used for the comprehensive evaluation of EdgeMap. We conducted all experiments on a system with an Intel i7-8700k CPU, 32 GB of RAM, and an NVIDIA RTX2080 GPU operating on Ubuntu 20.04. CARLsim [40] serves as our choice for the training and simulation of SNNs. In order to gauge performance, we employ the advanced, modified, cycle-accurate Noxim framework [43] to generate more accurate test data.

We leverage the software to simulate a hypothetical neuromorphic edge computing environment, using it as a benchmark to measure the performance of EdgeMap and other toolchains. The parameters of the target hardware are outlined in Table 1. The NCs in the edge devices are configured with 256 neurons and 64K synapses. The rest of the parameters are mentioned in Equations (6) and (10) and will be utilized in the experiments conducted throughout this paper.

### 5.2. Evaluated Applications

In this subsection, we delve into the efficiency evaluation of EdgeMap by considering various applications represented by SNNs. All applications are designed for edge computing and smaller edge devices, and are listed in Table 2; this table encapsulates vital details, including the input size, SNN connection topology, total neuron count, total synapses, and the whole spike count of the SNNs.

In this paper, we selected a suite of applications to evaluate EdgeMap. For classification tasks on the MNIST dataset [45], we used two different networks: MLP (multi-layer perceptron) and LeNet [46], both processing 28×28-pixel images (referred to as MNIST-MLP and MNIST-LeNet). In addition, we used an MLP network to process 28×28 pixel images from the Fashion MNIST dataset [47], referred to as Fashion-MNIST. The Heart Class application focuses on ECG-based heartbeat classification using images of 42×42 pixels [48]. For the CIFAR-10 classification task, we evaluated four different SNNs of varying complexity. These include LeNet [46] (CIFAR10-LeNet), AlexNet [49] (CIFAR10-AlexNet), VGG11 [50] (CIFAR10-VGG11), and ResNet [51] (CIFAR10-ResNet), all processing 32×32×3-pixel images. All of these applications are converted into spike-based models using the CNN-to-SNN conversion tool [52], and they are used to perform inference computations.

### 5.3. Evaluated Metrics

We evaluate the mapping toolchain for all SNN applications using the following metrics as listed below. These metrics are crucial as they directly reflect energy consumption and real-time processing capabilities, providing valuable insights into the system’s ability to meet the unique demands and constraints of edge computing scenarios.

- Energy consumption: This reflects the energy consumed by the neuromorphic hardware, a critical factor in edge device computing. It is modeled in Equation (6).
- Communication cost: This represents the cost of multi-chip or multi-devices edge computing scenarios. It is modeled in Equation (7).
- Throughput: This refers to the throughput of each application on the multi-core neuromorphic hardware. It is modeled in Equation (8).
- Average hop: Serves as an important indicator of communication efficiency in NoC-based neuromorphic hardware, which is modeled in Equation (9). We further analyze the maximum hop count across the mapping schemes to capture extremes.
- Average latency: This metric is indicative of the time delay in processing and is essential for time-sensitive edge computing applications. It is modeled in Equation (10); we also assess the maximum latency observed in the mapping schemes.
- Average congestion: This metric provides insight into the load balance across the network. It is modeled in Equation (11), and we extend our analysis to the maximum congestion experienced in the mapping schemes.
- Execution time: This metric measures the duration required for a mapping toolchain to produce a mapping result, encompassing both the partitioning and mapping stages.

### 5.4. Comparison Approaches

In our experiments, we conducted a comprehensive evaluation of various mapping toolchains, including our proposal, EdgeMap, which encompasses both the partitioning and mapping components. All of the toolchains we compare are summarized in Table 3. In these evaluations, we use SpiNeMap as a baseline for all metrics.

## 6. Results and Discussion

In this section, we present and discuss the results of our experiments, exploring the effectiveness of different mapping schemes in various applications. We evaluate their performance based on critical metrics, including energy consumption, communication cost, throughput, spike hop, spike latency, congestion, and execution time. These metrics are essential to comprehending the operational efficiency of each scheme under edge computing conditions.

### 6.1. Energy Consumption Analysis

**Figure 8:**
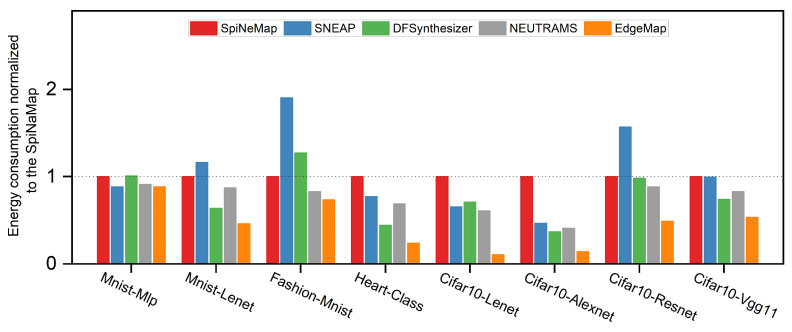
Normalized energy consumption analysis across different mapping schemes.

Figure 8 illustrates the energy consumption of different mapping schemes. As shown in the figure, EdgeMap demonstrates a clear advantage over other schemes in all applications, notably achieving an approximate 89% reduction in energy consumption compared to the baseline SpiNeMap in the CIFAR10-LeNet application. This considerable reduction is attributed to EdgeMap’s advanced optimization strategies that focus on energy consumption and communication costs during the mapping stage. It is worth mentioning that these optimization policies are not only unique to EdgeMap but also present a substantial advancement over similar strategies applied in previous work, such as the methods used in SpiNeMap, SNEAP, DFSynthesizer, and NEUTRAMS.

In relatively simple SNN structures used in the MNIST-MLP and Fashion-MNIST applications, all mapping schemes show comparably the same energy consumption. This might be a consequence of fewer neurons and connections involved in these applications. Nonetheless, even in these simple scenarios, EdgeMap still provides a notable improvement in energy consumption (12% decrease of SpiNeMap), which validates the effectiveness of its optimization techniques.

EdgeMap outperforms other mapping schemes in terms of average energy consumption, surpassing SpiNeMap by 57%, SNEAP by 66%, DFSynthesizer by 63%, and NEUTRAMS by 33%. This improvement is not incidental but is the result of EdgeMap’s specialized optimization policies, which are particularly designed for edge devices. It is important to note here that these policies include methods that significantly reduce the total number of spikes on the devices during the partitioning stage. This is a fundamental advancement over traditional methods and contributes significantly to the energy-saving performance of EdgeMap. Furthermore, the robustness of EdgeMap’s performance across a diverse range of SNNs with different scales shows EdgeMap’s generalizability to a broad array of SNN structures and edge devices.

In summary, EdgeMap’s excellence in managing energy consumption distinguishes it from other mapping schemes evaluated in this paper. Its superior efficiency makes it a compelling choice for edge devices, even when dealing with complex SNNs that have a large number of neurons and connections.

### 6.2. Communication Cost Analysis

**Figure 9:**
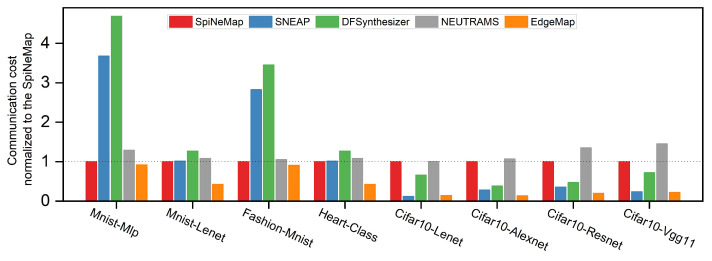
Normalized communication costs across different mapping schemes.

Figure 9 provides a comparison of the normalized communication costs across different mapping schemes, each measured relative to SpiNeMap. Clearly, EdgeMap emerges as the most effective scheme, indicating a significant improvement in reducing communication costs. In fact, when compared to SpiNeMap, SNEAP, DFSynthesizer, and NEUTRAMS, EdgeMap manages to reduce communication costs by 58%, 73.9%, 78.9%, and 65.8%, respectively. This substantial reduction is primarily the result of EdgeMap’s unique optimization approach, as outlined in Algorithm 1, which strategically modulates interactions among the neuron clusters. This technique represents an advancement over the existing methods employed by the compared schemes.

Moreover, our analysis shows that EdgeMap’s performance improvement is not constant but scales proportionally with the complexity of the SNNs. That is, the larger the scale of the SNNs, the more pronounced the performance benefits of our optimization algorithm. This correlation suggests that EdgeMap’s optimization strategies are well-suited to complex SNNs that typically pose challenges for existing mapping schemes. This adaptive nature of performance enhancement is of great importance in an edge computing environment. It ensures that the deployment of complex SNNs does not translate into excessive communication overhead. It is worth noting that this advantage is not merely theoretical, but it has been validated across various scales of SNNs and in different edge computing scenarios, reinforcing the robustness and generalizability of our results.

In conclusion, the performance advantage demonstrated by EdgeMap in terms of communication cost reduction is a testament to the effectiveness of its novel optimization approach. This not only separates EdgeMap from other mapping schemes but also underscores its suitability for complex SNNs in an edge computing environment.

### 6.3. Throughput Analysis

**Figure 10:**
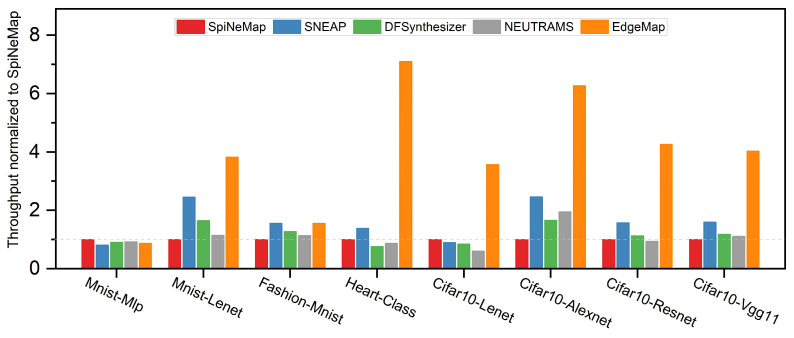
Normalized throughput across different mapping schemes.

Figure 10 offers a comparative analysis of the normalized throughput for each application across the mapping schemes under evaluation, with each scheme normalized to SpiNeMap. This comparison is pivotal for understanding the operational efficiency of each scheme within the edge computing context. The analysis reveals that EdgeMap surpasses all other mapping schemes in terms of throughput performance. Notably, EdgeMap’s throughput is 4.02× greater than that of SpiNeMap, and outperforms SNEAP, DFSynthesizer, and NEUTRAMS by 2.52×, 3.43×, and 3.65×, respectively.

The remarkable throughput advantage of EdgeMap is primarily due to its focus on multi-objective optimization during the mapping process, as detailed in Algorithm 2. This distinguishing factor sets EdgeMap apart from the other schemes, which do not typically incorporate this aspect into their mapping methodologies. By focusing on multi-objective optimization, EdgeMap can effectively harness the computational capabilities of edge devices, facilitating a significant improvement in throughput, satisfying the real-time processing requirement. As the complexity of the SNNs increases, the throughput advantage of EdgeMap becomes more pronounced, suggesting that our toolchain is particularly suited for large-scale, complex SNN applications.

Overall, these results affirm EdgeMap’s effectiveness in delivering superior throughput performance and its scalability in handling complex SNNs, making it a highly competitive solution in the field of SNN mapping on edge devices.

### 6.4. Spike Hop Analysis

**Figure 11:**
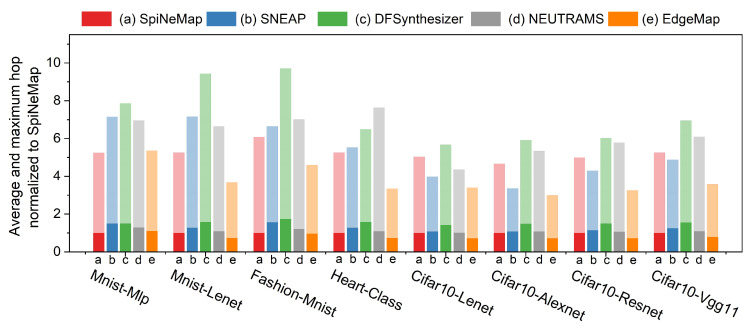
Normalized average and maximum hop analysis across different mapping schemes.

Figure 11 presents the normalized average and maximum hops for each application under each of the evaluated mapping schemes, normalized to SpiNeMap. A single color represents each mapping scheme, the darker shade indicates the average hop, and the lighter shade denotes the maximum hop. The hop count serves as a crucial indicator of collaborative capability in edge computing scenarios.

EdgeMap consistently outperformed all other mapping schemes across all applications, highlighting its benefits in edge computing scenarios. Specifically, EdgeMap’s average hop count is 19.4% lower than that of SpiNeMap, and its maximum hop count is 29.9% lower. Moreover, when compared with SNEAP, DFSynthesizer, and NEUTRAMS, EdgeMap reduces the average hop count by 36.5%, 48.1%, and 27.3%, respectively, while its maximum hop count is decreased by 27.8%, 48.1%, and 41.9%.

With the scaling of the SNNs, both average and maximum hops increase correspondingly. However, EdgeMap effectively keeps this increase within a manageable range, primarily due to the mapping strategy deployed in Algorithm 2, which emphasizes the spatial proximity of interconnected neurons. This approach curbs the exponential growth of hop counts as the network expands, thereby contributing significantly to EdgeMap’s efficient performance within edge computing environments. Notably, this control over hop counts is instrumental in reducing latency, making EdgeMap an ideal choice for real-time applications where responsiveness is key.

In summary, the results underscore EdgeMap’s efficacy in maintaining efficient communication among neurons in large-scale SNN applications, resulting in lower latency, and making it a compelling choice for edge computing scenarios.

### 6.5. Spike Latency Analysis

**Figure 12:**
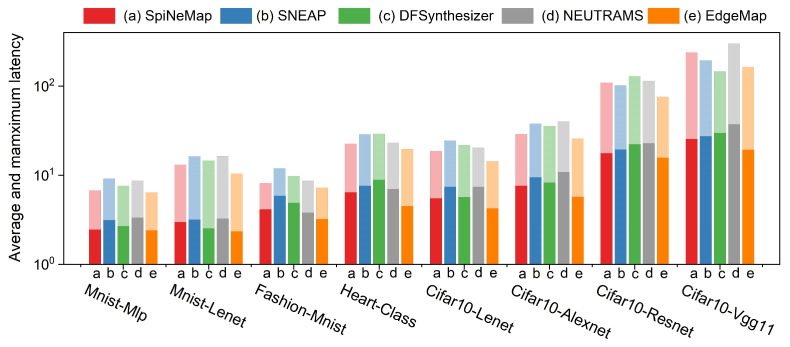
Average and maximum latency analysis across different mapping schemes.

Figure 12 illustrates the comparative analysis of both average and maximum spike latencies across applications for the evaluated mapping schemes. Consistent color patterns are used to denote individual mapping schemes, with darker shades representing average latency, and lighter shades indicating maximum latency. Our evaluation reveals that EdgeMap consistently outperforms other schemes, demonstrating lower average and maximum latencies. Specifically, EdgeMap’s average latency is 19.8% lower than that of SpiNeMap, while its maximum latency is reduced by 22.5%. Comparatively, EdgeMap’s average latency reduces by 33.4%, 29.2%, and 35.5% against SNEAP, DFSynthesizer, and NEUTRAMS, respectively. Similarly, its maximum latency is lower by 28.6%, 22.9%, and 33.7%.

The latency in each application arises from two components: the processing time for spikes and the spike communication latency. As the scale of the SNN increases, so does the hardware scale and, thus, the transmission time, leading to increased latency. However, EdgeMap’s optimization of communication costs effectively reduces congestion, resulting in a decrease in latency.

In summary, EdgeMap demonstrates superior performance by consistently maintaining lower latency levels across all applications. This is primarily achieved through its strategic optimization, which effectively mitigates congestion, enabling EdgeMap to maintain optimal performance even as the network scales. These features underscore EdgeMap’s suitability for edge computing environments, where low latency is crucial.

### 6.6. Congestion Analysis

**Figure 13:**
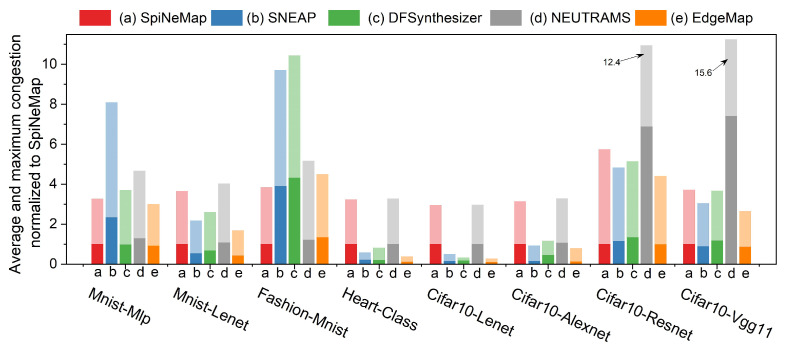
Normalized average and maximum congestion analysis across different mapping schemes.

Congestion is a crucial metric that reflects the collaborative capability of edge computing scenarios, and as such, it plays a pivotal role in our analysis. As Figure 13 demonstrates, EdgeMap consistently outperforms other mapping schemes across all applications, exhibiting superior performance. Each mapping scheme in the figure is represented by a specific color, with darker shades indicating average congestion and lighter shades representing maximum congestion.

Specifically, EdgeMap outperforms other mapping schemes across all applications. Specifically, the average congestion of EdgeMap is 39.5% lower than that of SpiNeMap, while its maximum congestion is reduced by 40.8%. Furthermore, compared to SNEAP, DFSynthesizer, and NEUTRAMS, EdgeMap’s average congestion decreases by 47.8%, 42.6%, and 81.2% respectively, with maximum congestion reduced by 47.5%, 43.7%, and 74%. Interestingly, NEUTRAMS exhibits inferior performance, which can be attributed to its methodology. This scheme optimizes the communication cost only during the partitioning stage, mapping neuron clusters onto devices sequentially. As a result, it suffers from high congestion.

In summary, EdgeMap is shown to be a highly effective mapping scheme, significantly reducing congestion in edge computing applications. This finding underlines EdgeMap’s capabilities in boosting performance and efficiency in edge scenarios, providing valuable insights for researchers in the field.

### 6.7. Execution Time Analysis

**Figure 14:**
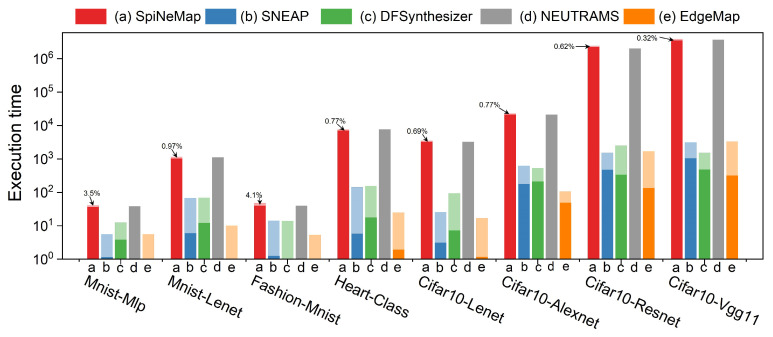
Execution times across different mapping schemes.

Figure 14 provides a comparison of the execution time across different applications for the evaluated mapping schemes. We divided the total execution time into two stages: the partitioning stage and the mapping stage. A uniform color scheme is employed to represent each mapping scheme, with darker shades indicating the partition time and lighter shades denoting the mapping time.

Interestingly, the execution times of SpiNeMap and NEUTRAMS are relatively similar, which can be attributed to their identical partitioning algorithms and objectives. However, NEUTRAMS does not incorporate a separate mapping stage. The partition time dominates the execution time, accounting for 96.5% of the total in SpiNeMap and rising to 99.68% for the CIFAR10-VGG11 application as the complexity of SNNs increases.

EdgeMap’s execution process differs significantly from other schemes, with the mapping stage accounting for a considerable portion of the total execution time. For the MNIST-MLP network, the mapping time essentially occupies the entire execution time. As the complexity of the SNNs increases, so does the time spent on mapping. For instance, the mapping time constitutes 90.3% of the total execution time for the CIFAR10-VGG11 network. Furthermore, EdgeMap outperforms SpiNeMap by an impressive factor of 1225.44× in the average execution time. The partitioning stage and the mapping stage are faster by 11268.9× and 131×, respectively. Despite having a comparable total execution time to SNEAP, EdgeMap demonstrates superior efficiency, with partition times being 3.36× faster and mapping times 0.82× as fast. When compared with DFSynthesizer and NEUTRAMS, EdgeMap’s average total execution time is more efficient, specifically 0.94× and 1113×, respectively. The partition time for DFSynthesizer is 2.1× longer, further emphasizing EdgeMap’s efficacy.

## 7. Conclusions

In this paper, we introduced EdgeMap, an optimized toolchain for mapping SNNs onto neuromorphic hardware within edge computing scenarios. EdgeMap demonstrates significant improvements in energy efficiency, communication cost reduction, and throughput enhancement. It also successfully minimizes both spike latency and congestion, highlighting its superior real-time performance, which is crucial for edge applications. Through a two-stage mechanism, partitioning and mapping, EdgeMap ensures rapid execution speed and is highly adaptable to edge computing conditions. Remarkably, its robust performance remains consistent even as the network complexity escalates, reinforcing EdgeMap’s supremacy over other contemporary mapping methods.

Despite its significant advantages, EdgeMap still has its limitations. Currently, our SNN mapping is largely based on static optimization, concentrating primarily on fixed network structures. However, edge computing scenarios often require dynamic adjustments in response to environmental changes or task requirements. This dynamic aspect of edge computing presents a challenge that EdgeMap, in its current form, may not handle directly. Additionally, we make an assumption that all edge devices have equal computational power and overlook geographical factors. This assumption may not reflect real-world scenarios. Variations in hardware can cause differences in the computational power of edge devices. Similarly, the geographical location can significantly affect communication latency and data transfer efficiency. These factors could increase the complexity of mapping, indicating an area where our EdgeMap method may require enhancements.

Moving forward, our goal is to incorporate more flexible optimization objectives within the EdgeMap toolchain to accommodate the needs of increasingly complex computing environments. We also aim to explore methods for mapping SNNs with online learning capabilities onto edge devices. In conclusion, EdgeMap shows promise as a strategy for efficiently mapping SNNs onto edge devices. Although it has demonstrated exceptional results, improvements and adaptations are necessary to reflect real-world edge computing scenarios more accurately.

## Figures and Tables

**Table 1:** Parameters of the target neuromorphic edge devices.

Parameter	Neurons/Core	Synapses/Core	$e_{s}$	$e_{w}$	$l_{s}$	$l_{w}$
Value	256	64k	1	0.1	1	0.01

**Table 2:** Application used for the evaluation of EdgeMap.

Applications	MNIST-MLP	MNIST-LeNet	Fashion-MNIST	Heart Class	CIFAR10-LeNet	CIFAR10-AlexNet	CIFAR10-VGG11	CIFAR10-ResNet
	28×28×1	28×28×1	28×28×1	42×42×1	32×32×3	32×32×3	32×32×3	32×32×3
Topology	MLP ^1^	CNN ^2^	MLP ^3^	CNN ^4^	CNN ^5^	CNN ^6^	CNN ^7^	CNN ^8^
Neuron number	1193	7403	1393	17,001	11,461	794,232	9,986,862	9,675,543
Synapses	97,900	377,990	444,600	773,112	804,614	39,117,304	47,737,200	48,384,204
Total spikes	358,000	1,555,986	10,846,940	2,209,232	7,978,094	574,266,873	796,453,842	5,534,290,865

^1^ Feedforward(784-100-10). ^2^ Conv((5,5),(1,1),6)-AvgPool(2,2)-Conv((5,5),(1,1),16)-AvgPool(2,2)-FC(500)-FC(10). ^3^ Feedforward(784-500-100-10). ^4^ Conv((5,5),(1,1),6)-AvgPool(2,2)-Conv((5,5),(1,1),16)-AvgPool(2,2)-FC(10). ^5^ Conv((5,5),(1,1),6)-AvgPool(2,2)-Conv((5,5),(1,1),16)-AvgPool(2,2)-FC(500)-FC(10). ^6^ Conv((11,11),(4,4),96)-Maxpool(2,2)-Conv((5,5),(1,1),256)-Maxpool(2,2)-Conv((3,3),(1,1),384)-Conv((3,3),(1,1),256)-Maxpool(2,2)-FC(4096-4096-10). ^7^ Conv((3,3),(1,1),64)-MaxPool(2,2)-Conv((3,3),(1,1),128)-MaxPool(2,2)-Conv((3,3),(1,1),256)-Conv((3,3),(1,1),256)-MaxPool(2,2)-Conv((3,3),(1,1),512)-Conv((3,3),(1,1),512)-MaxPool(2,2))-Flatten-FC(4096-4096-10). ^8^ Conv((3,3),(1,1),64)-MaxPool(2,2)-Conv((3,3),(1,1),128)-MaxPool(2,2)- Conv((3,3),(1,1),256)-Conv((3,3),(1,1),256)-MaxPool(2,2)-Conv((3,3),(1,1),512)-Conv((3,3),(1,1),512)-MaxPool(2,2)- FC(4096-4096-10).

**Table 3:** Comparison of different mapping toolchains.

Toolchains		SpiNeMap [13]	SNEAP [22]	DFSynthesizer [23]	NEUTRAMS ${}^{4}$ [21]	EdgeMap
Partition	Algorithm	Kernighan–Lin(KL)	METIS	Greedy algorithm ${}^{1}$	Kernighan–Lin(KL)	Streaming-based
	Objective	Communication cost	Communication cost	Resource Utilization.	Communication cost	Communication cost
Mapping	Algorithm	PSO	SA ${}^{2}$	LSA ${}^{3}$	–	NSGA-II
	Objective	Communication cost	Average Hop	Energy consumption	–	Communication cost & Energy consumption

^1^ This algorithm is a modified greedy algorithm. ^2^ SA is short for simulated annealing. ^3^ LSA is short for the local search algorithm. ^4^ In NEUTRAMS, the mapping stage is not mentioned, we use sequential mapping as the default.

## Data Availability

Data will be made available upon reasonable request to the authors.
